# Direct maximum parsimony phylogeny reconstruction from genotype data

**DOI:** 10.1186/1471-2105-8-472

**Published:** 2007-12-05

**Authors:** Srinath Sridhar, Fumei Lam, Guy E Blelloch, R Ravi, Russell Schwartz

**Affiliations:** 1 Computer Science Department, Carnegie Mellon University, Pittsburgh, PA, USA; 2 Computer Science Department, Brown University, Providence, RI, USA; 3 Tepper School of Business, Carnegie Mellon University, Pittsburgh, PA, USA; 4 Department of Biological Sciences, Carnegie Mellon University, Pittsburgh, PA, USA

## Abstract

**Background:**

Maximum parsimony phylogenetic tree reconstruction from genetic variation data is a fundamental problem in computational genetics with many practical applications in population genetics, whole genome analysis, and the search for genetic predictors of disease. Efficient methods are available for reconstruction of maximum parsimony trees from haplotype data, but such data are difficult to determine directly for autosomal DNA. Data more commonly is available in the form of genotypes, which consist of conflated combinations of pairs of haplotypes from homologous chromosomes. Currently, there are no general algorithms for the direct reconstruction of maximum parsimony phylogenies from genotype data. Hence phylogenetic applications for autosomal data must therefore rely on other methods for first computationally inferring haplotypes from genotypes.

**Results:**

In this work, we develop the first practical method for computing maximum parsimony phylogenies directly from genotype data. We show that the standard practice of first inferring haplotypes from genotypes and then reconstructing a phylogeny on the haplotypes often substantially overestimates phylogeny size. As an immediate application, our method can be used to determine the minimum number of mutations required to explain a given set of observed genotypes.

**Conclusion:**

Phylogeny reconstruction directly from unphased data is computationally feasible for moderate-sized problem instances and can lead to substantially more accurate tree size inferences than the standard practice of treating phasing and phylogeny construction as two separate analysis stages. The difference between the approaches is particularly important for downstream applications that require a lower-bound on the number of mutations that the genetic region has undergone.

## Background

The sequencing of the human genome has made it possible to conduct genome-wide studies on genetic variations in human populations. Most of these variation data are in the form of single nucleotide polymorphisms (SNPs), single DNA bases that have two common variants in a population, of which several million have now been identified [1]. Phylogenetic inference is central to identifying shared ancestry among populations and is also useful as a means of increasing the statistical power of association studies used to detect disease-related variations [2]. Furthermore, phylogenies can provide specific guidance as to the selection of marker SNPs for such studies, for example by allowing one to avoid variant sites that have appeared multiple times in an evolutionary tree and that are therefore likely to confound association tests. Phylogenetics on short evolutionary time scales, such as within a single species, is generally performed using a maximum parsimony objective [3], i.e., finding trees that explain the observed data with the minimum possible number of mutations. On such data, it is usually assumed that one must find a *Steiner tree *in which observed sequences may be present anywhere in the tree and additional *Steiner nodes *may be introduced. This is in contrast to the species trees used to describe longer time scales, where observed sequences are generally found only at the leaves of the tree. Although inferring maximum parsimony Steiner trees on binary SNP data (haplotypes) is an NP-hard problem [4], there are excellent methods now available for solving it in practice, including fast heuristics suitable for difficult instances [5-8], fixed parameter tractable methods for provably efficient optimal solutions in some cases [9,10], and integer linear programming methods for provably optimal solutions of many harder cases [11].

Unfortunately, the haplotype input data these methods assume, also known as "phased" data, are not easily available for autosomal genetic regions. Large-scale genetic studies usually instead must gather unphased, or genotype, data, in which haplotype contributions from two homologous chromosomes are conflated with one another.

**Figure 1:**
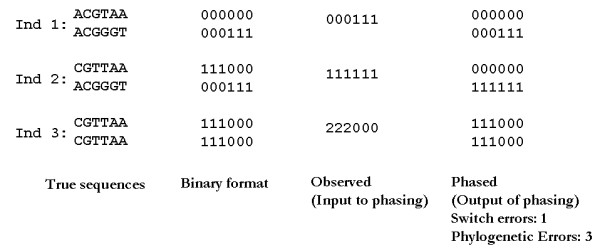
**Phasing: computationally inferring genotypes from haplotypes**. Although DNA sequences consist of four bases, single nucleotide polymorphisms (SNPs) are biallelic. Therefore, the sequence variation can be expressed using binary symbols. The observed genotype sequences consist of conflated combinations of two true haplotype sequences. Programs that computationally infer haplotypes attempt to minimize switch errors.

To illustrate the problem, it will be helpful to arbitrarily denote the minor allele at each SNP site by 1 and the major allele by 0. In a genotype data set, we only observe the number of minor alleles present at each SNP site, which we will denote by 0 for homozygous major, 1 for heterozygous and 2 for homozygous minor. For example, see Figure 1. Hence, if we examine *m *sites, then a genotype sequence is a string of the form {0, 1, 2}^*m *^while a haplotype sequence is a string of the form {0, 1}^*m*^. A pair of haplotype sequences is *consistent *with (explains) a genotype sequence when they have the same allele counts at all sites. In the {0, 1, 2} notation above, a pair of haplotypes is consistent with a genotype when the sum of the two haplotype vectors produces the genotype vector.

**Figure 2:**
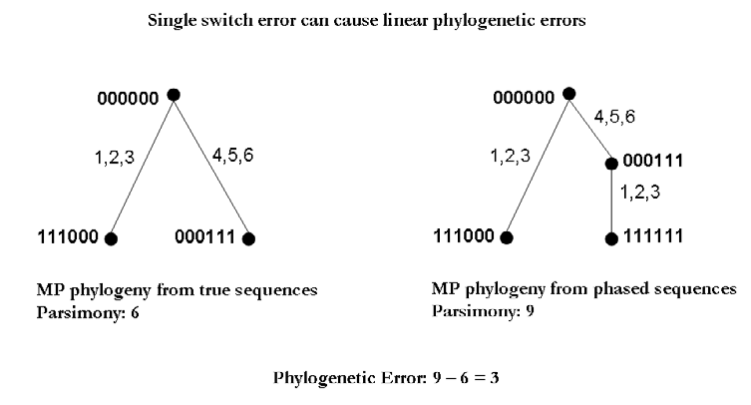
**Phylogenetic error**. Although switch errors in phase inference can be small, in this case 1, the phylogeny size could be significantly altered. Therefore estimates such as mutation rates could be significantly affected if performed on computationally inferred haplotypes as opposed to genotypes. Moreover, in current methods it is impossible to say if the inferred phylogeny size is larger or smaller than that of the phylogeny from the true haplotypes.

While mitochondrial and Y chromosome data can serve for tracking population histories on broad scales [12], autosomal phylogenies are still independently valuable for practical applications in association study design, marker selection, and the identification of specific variant sites that are unusually mutable, repeatedly altered by gene conversion, or under selective pressure to recurrently mutate. Phylogeny inference cannot generally be performed directly on genotype data and in practice one must therefore analyze autosomal data by first computationally phasing the data to predict the haplotypes [13]. Many methods are now available for this phasing problem, such as PHASE [14], fastPHASE [15], HAP [16] and PPH [17]. This phasing step, however, can produce erroneous assignments and the maximum parsimony phylogeny on the computationally phased genotypes need not be the same as, or even close to, the maximally parsimonious tree consistent with the original unphased genotypes. Phasing programs are typically designed to minimize the "switch error," in which the contributions from two homologous chromosomes are swapped between two consecutive markers (see [15] for the formal definition). Yet a single switch error in a phasing assignment can introduce a large number of errors (linear in the number of markers) in the resulting phylogeny assignment, as shown in Figures 1 and 2. Even high-quality phasing methods can thus produce poor-quality phylogenies.

A limited amount of prior work has examined the prospect of inferring maximum parsimony phylogenies directly from genotype data. Notice that in such problems, we wish to determine a pair of haplotypes for each input genotype sequence such that the maximum parsimony phylogeny size on the set of haplotypes is minimized. Gusfield showed that the problem can be efficiently solved when the genotype data are consistent with a perfect phylogeny [17], an evolutionary tree in which each variant site mutates only once. Several subsequent algorithms were developed for the same problem that were either simpler or faster asymptotically [17-21]. While the perfect phylogeny assumption is restrictive, this variant does have practical importance as a technique for fast phasing (e.g., [16]). The perfect phylogeny assumption will not be true in general, however. In particular, it will not allow analysis of data containing recurrently mutating sites, the detection of which is an important reason for studying phylogenetics of autosomal DNA. Halperin et al. [16] generalized Gusfield's perfect phylogeny method heuristically to allow limited solution of phylogenies deviating slightly from the assumption of perfection. These are called *near-perfect phylogenies *[22] and specifically *q-near-perfect *(or *q-imperfect*) when *q *additional mutations are needed beyond those required to produce a perfect phylogeny. Song *et al.*[23] and Sridhar *et al.*[24] developed rigorous methods for efficiently finding maximum parsimony near-perfect phylogenies, but these methods proved practical only for small *q *(at most 2). In practice, the problem of finding maximum parsimony phylogenies from genotype data has remained intractable for all but the simplest data sets.

We note that the parsimony based approach described above is different from finding haplotypes corresponding to the given genotypes based on 'pure parsimony,' an objective that minimizes the number of distinct haplotypes needed to explain the observed genotypes as opposed to minimizing the number of mutations. The pure parsimony problem is NP-complete as well and there are integer program based approaches that solve problem instances of reasonable size [25]. Pure parsimony and maximum parsimony phylogenetic trees share some properties that we can exploit in our method. The solution to the pure parsimony problem provides a lower bound on the size of the maximum parsimony phylogeny, as no phylogeny can have fewer mutations than one less than the minimum number of haplotypes needed to explain the genotypes. Furthermore, the solution of the pure parsimony problem also provides a good starting set of haplotypes from which we can obtain an upper-bound for the size of the maximum parsimony phylogeny.

In this paper, we provide the first general, practical methods for maximum parsimony phylogeny inference from genotypes and use these methods to assess the inaccuracies introduced by phasing genotypes prior to phylogeny inference. Our algorithm relies on solving integer linear programs and allows for efficient solution of moderate-sized problem instances but large imperfection. As an immediate application, our method can be used to infer the minimum number of recurrent mutations required to explain the given set of genotypes. We apply the resulting methods to a selection of real and simulated data, where we compare the true imperfection, imperfection from haplotypes computationally inferred from genotypes and imperfection directly obtained from genotypes. This analysis shows that the phasing step often increases inferred phylogeny size, overestimating the true maximum parsimony. Motivated by our observations, we introduce a new *phylogenetic error *statistic that is better suited for assessing phase accuracy for phylogenetic applications than the standard switch error statistic [15].

## Results and Discussion

We now present the results of a series of empirical tests to assess the utility of the method on real and simulated genetic data. With both kinds of data, we begin with known haplotypes and then artificially pair them to produce genotypes. For each problem instance, we reconstruct maximum parsimony (MP) phylogenies in three ways: directly from the genotypes using the algorithm presented in this paper, from the original (true) haplotypes and from haplotypes computationally inferred from the genotypes using fastPHASE [15] and haplotyper [26], two leading methods for haplotype inference. We use the notation *T*_*min*_, *T*_*true*_, and *T*_*phase *_to denote the MP phylogeny from the genotypes, true haplotypes and inferred haplotypes (either using fastPHASE or haplotyper) respectively. We further denote the parsimony score (number of mutations) of a phylogeny *T *by length(*T*). For phylogeny *T *that is either *T*_*phase *_or *T*_*min*_, we define a *phylogenetic error *based on length(*T*_*true*_) as follows.

### Definition 1

*The *phylogenetic error *of Phylogeny T (T*_*min *_*or T*_*phase*_*) is *|length(*T*_*true*_) - length(*T*)|. *Phylogeny T is said to have a positive error if *length(*T*) > length(*T*_*true*_) *and negative error if *length(*T*) < length(*T*_*true*_).

Note that it is impossible for *T*_*min *_to have positive phylogenetic error. This is because our algorithm optimizes over all possible haplotypes consistent with the given set of genotypes and selects the one that minimizes the size of the phylogenetic tree. In contrast, *T*_*phase *_can suffer from both types of errors and it is impossible to know if the size of the true phylogeny is larger or smaller than *T*_*phase*_. The following definition of an *imperfection *of a phylogeny has been widely used.

### Definition 2

*The *imperfection *of a phylogeny T constructed for an input set of sequences (genotypes or haplotypes) with m varying sites is *length(*T*) - *m*.

Simply stated, the imperfection is the minimum number of *recurrent *mutations required to explain the sequences using the phylogeny. Notice that if there are *m *varying sites in an input set of genotypes then every possible set of haplotypes that explain it must have *m *varying sites as well. The experiments presented in the following section allow us to understand the gap between the size of the phylogeny from genotypes, the true size and the artificially inflated sizes due to incorrect phase inference.

### Simulated Data

**Figure 3:**
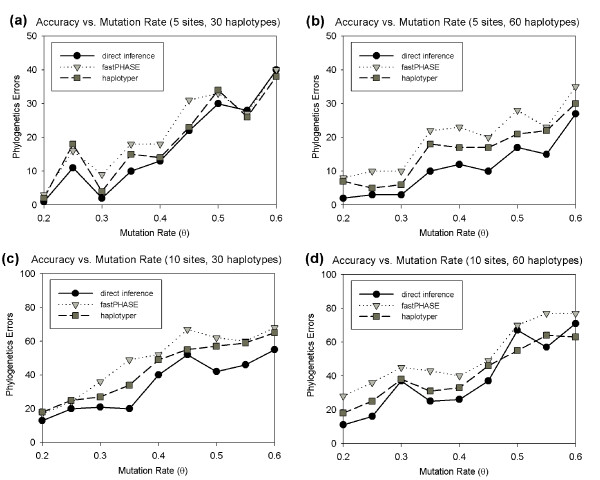
**Phylogenetic error as a function of mutation rate for varying dataset sizes**. Each plot shows the phylogenetic errors for inferences from our direct inference method (black circles), indirect inference using fastPHASE (light grey triangles), and indirect inference using haplotyper (dark grey squares) as a function of the mutation rate *θ*. Plots are provided for two window sizes (5 and 10 SNPs) and for two population sizes (30 and 60 haplotypes). Each data point in the plot was computed by running each algorithm over 200 randomly generated data-sets, (a) window size 5, 30 haplotypes. (b) window size 5, 60 haplotypes. (c) window size 10, 30 haplotypes. (d) window size 10, 60 haplotypes.

Due to difficulty of obtaining phase-known autosomal data, we begin by examining simulated data. We used coalescent simulations to generate recombination-free haplotypes and genotypes for varying mutation rates and used these for a series of tests on how the accuracy of our method and the two comparative haplotype-based approaches varied with different parameter values. Each test measured the total number of errors of each method in 200 independently generated data sets. We first varied the mutation rate parameter *θ *to test its influence on the accuracy of all the methods. The results are provided in Figure 3. We find that the relative performance of the three methods is fairly consistent. The greatest number of errors is generally made by fastPHASE and the least by direct phylogeny inference from the genotypes, with haplotyper in between. As one would expect, the number of errors of all three methods increases with increasing mutation rate. The curves are not monotonic, but additional simulation runs identical to those described here (data not shown) show no conservation of specific peaks and troughs of the graphs, indicating that they reflect only random noise due to a high variance in phylogenetic errors across trials. Table 1 separates the results of the two indirect methods, fastPHASE and haplotyper, into positive and negative errors. Both methods show mixtures of generally similar numbers of positive and negative phylogenetic errors with no apparent consistent trends towards favoring one or the other error type as one particular parameter varies. Note that by definition, our new method cannot produce positive errors and all errors it produces therefore reflect underestimates of phylogeny size.

**Table 1:** Positive and negative errors for indirect phylogeny inference with varying mutation rate

SNPs	*n*_*H*_	Method	*θ *= 0.20	0.25	0.30	0.35	0.40	0.45	0.50	0.55	0.60
5	30	fastPHASE positive errors	2	8	8	13	7	15	14	8	14
5	30	fastPHASE negative errors	1	8	1	5	11	16	19	19	26
5	30	haplotyper positive errors	1	10	2	10	3	6	12	5	12
5	30	haplotyper negative errors	1	8	2	5	11	17	22	21	26

10	30	fastPHASE positive errors	7	12	27	38	28	38	38	27	42
10	30	fastPHASE negative errors	10	12	9	11	24	29	24	23	26
10	30	haplotyper positive errors	7	14	15	23	28	24	37	41	40
10	30	haplotyper negative errors	11	11	12	11	21	31	20	18	25

5	60	fastPHASE positive errors	6	7	7	15	16	13	16	11	14
5	60	fastPHASE negative errors	2	3	3	7	7	7	12	12	21
5	60	haplotyper positive errors	5	3	3	9	7	8	7	7	6
5	60	haplotyper negative errors	2	2	3	9	10	9	14	15	24

10	60	fastPHASE positive errors	24	25	25	29	28	32	43	54	41
10	60	fastPHASE negative errors	4	11	20	14	12	17	27	23	36
10	60	haplotyper positive errors	11	13	14	13	22	25	27	42	34
10	60	haplotyper negative errors	7	12	24	18	11	21	28	22	29

The table separates the phylogenetic errors from the experiments of Figure 2 into positive and negative errors for indirect phylogeny inference using fastPHASE and haplotyper.

**Figure 4:**
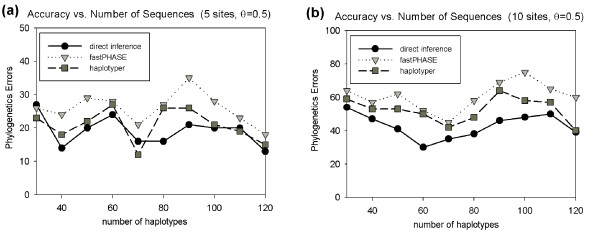
**Phylogenetic error as a function of population size**. Each plot shows the phylogenetic errors for inferences from our direct inference method (black circles), indirect inference using fastPHASE (light grey triangles), and indirect inference using haplotyper (dark grey squares) as a function of number of input haplotypes. Plots are provided for two window sizes (5 and 10 SNPs). Each data point in the plot was computed by running each algorithm over 200 randomly generated data-sets, (a) window size 5. (b) window size 10.

We next tested variation in accuracy with the number of haplotype sequences sampled for fixed mutation rate with *θ *= 0.5. The results are shown in Figure 4. Our direct methods show a slightly more pronounced advantage for 10-SNP windows than 5-SNP windows. This could simply be due to higher variance in the results of the 5-SNP windows. Table 2 shows the breakdown of the indirect methods into positive and negative errors, with both indirect methods again showing a mixture of comparable numbers of positive and negative errors across the parameter range, haplotyper again shows generally better accuracy than fastPHASE by this measure. One might expect that with increase in the number of haplotypes, the number of mutations required to explain the data would increase as well. Therefore, the number of errors should increase with the number of haplotypes. This, however, does not seem to be the case in practice, an observation that can be explained by the fact that greater numbers of haplotypes provides more information and thus yield improved accuracy in phase inference. Therefore, the number of phylogenetic errors roughly stay the same with the increase in the number of haplotypes for all the methods.

**Table 2:** Positive and negative errors for indirect phylogeny inference with varying sample sizes

SNPs	Method	*n*_*H *_= 30	40	50	60	70	80	90	100	110	120
5	fastPHASE positive errors	10	12	15	14	11	15	25	15	10	10
5	fastPHASE negative errors	16	12	14	14	10	12	10	13	13	8
5	haplotyper positive errors	5	6	7	10	3	11	12	7	5	4
5	haplotyper negative errors	18	12	15	17	9	15	14	14	14	11

10	fastPHASE positive errors	38	32	43	34	31	41	46	53	41	38
10	fastPHASE negative errors	26	25	19	18	14	17	23	22	24	22
10	haplotyper positive errors	32	26	31	28	35	23	36	30	23	14
10	haplotyper negative errors	27	27	22	22	17	25	28	28	34	26

The table separates the phylogenetic errors from the experiments of Figure 3 into positive and negative errors for indirect phylogeny inference using fastPHASE and haplotyper.

### Mitochondrial DNA

The next step in our analysis used mitochondrial DNA (mtDNA), which is naturally haploid. Although one would not normally need to phase mitochondrial DNA, we use it in our validation because it provides a source of large numbers of known haplotypes and because it provides a good model of recombination-free DNA. The lack of recombination in the mitochondrial DNA means that if the most parsimonious phylogeny on the genotypes is *q*-imperfect, then that region must have undergone a minimum of *q *recurrent mutations. The mitochondrial genome contains known regions of high mutation rate that allow us to validate the ability of phylogenetic imperfection to identify true sites of recurrent mutation, a key application of our method. For the purpose of these tests, we generated artificial diploids by randomly combining 60 mitochondrial complete sequences (16,569 bases) from a data set of Fraumene *et al.*[27] to produce thirty diploids. We then computationally inferred haplotypes from the all of the genotypes using fastPHASE. Haplotyper was omitted from these tests because the data set was larger than it could process. We then constructed phylogenies for all sliding windows of 50 bases across the data set by each of three methods: maximum parsimony using true haplotypes, inferred haplotypes and directly from the genotypes. Our method required 116 seconds on a desktop Linux PC to reconstruct the phylogenies for all the sliding windows, clearly demonstrating its practical efficiency.

**Figure 5:**
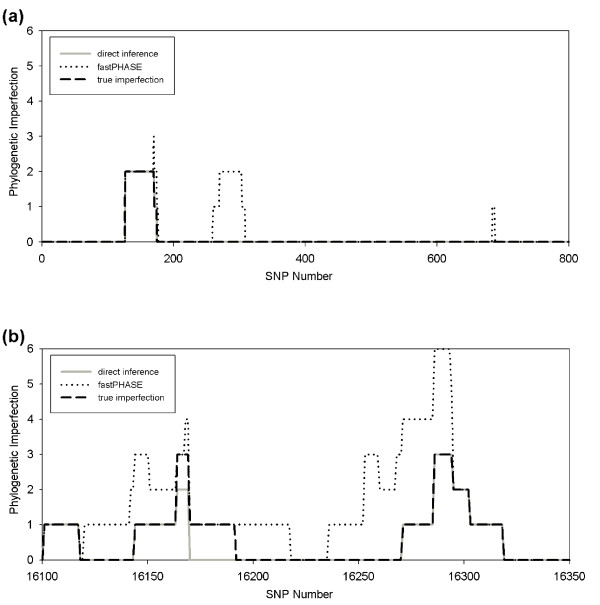
**Mitochondrial DNA D-loop**. Imperfection around two high-variation segments (bp 1:800 and 16100:16350) of the D-loop of the mtDNA. Each position on the x-axis denotes the central nucleotide of the window examined. The y-axis shows the inferred imperfection by our direct method (solid grey line), imperfections inferred by the indirect method using fastPHASE (dotted black line), and the true imperfection (dashed black line), (a) bp 1 to 800. (b) bp 16100 to 16350.

Figure 5 shows the results for two regions of the mitochondrial D-loop that are known to have unusually high mutation rates [28]. The intervening sequence between these two regions, where mutation rate is low, is not shown since all windows have true imperfection zero. The genotype imperfection is identical to the true imperfection for the large majority of windows (zero positive and negative phylogenetic errors). While inferences from genotypes could err in the direction of underestimating the true haplotype imperfection, they nonetheless appear in practice to provide very good estimates of the true imperfection on these data. Genotype imperfection is never less than one below the true imperfection, i.e., at most 1 negative phylogenetic error for any window. Imperfection from inferred haplotypes is usually higher than the true imperfection in the imperfect regions, often substantially so, demonstrating that incorrect phasing can lead to large phylogenetic errors.

### Phase-known Autosomal DNA

Only a very limited amount of true phase-known autosomal data is available. We chose to examine a set taken from the lipoprotein lipase (LPL) gene [29], which is the only true phase-known data publicly available that has a sufficiently large population sample and number of SNPs to provide a challenging test for the methods considered here. The dataset consists of 144 haplotypes (77 distinct) belonging to three different ethnicities and 86 SNPs. The true genotypes corresponding to the haplotypes were not published for this data sets and so we duplicated the first haplotype to obtain 78 distinct sequences and then randomly paired them to produce 39 artificial genotypes from the true haplotypes. As in the previous case, we ran fastPHASE and haplotyper on all of the SNPs put together to obtain inferred haplotypes. Unlike mtDNA, the autosomal chromosomes undergo recombinations and so we used the HAP webserver [16] to break the 86 SNPs into blocks. We obtained 22 blocks which we assume to be recombination-free. We then estimated the size of the phylogenies within each of the blocks separately from the true haplotypes, inferred haplotypes and genotypes directly. Note that we would expect this to be a particularly difficult dataset for our algorithm because haplotyper and fastPHASE made inferences from all the SNPs at once, whereas our method was run on each block independently.

**Figure 6:**
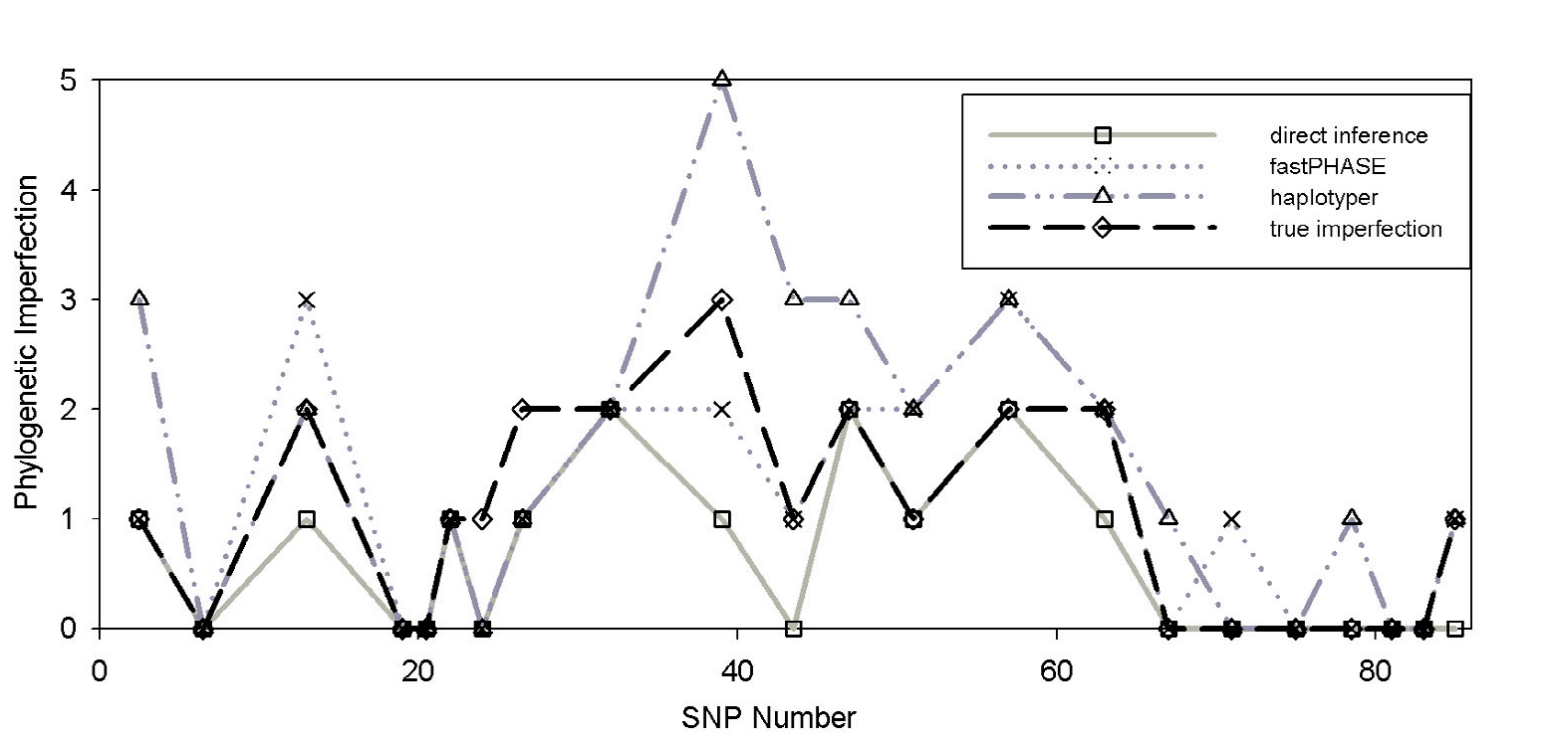
**Phylogenetic errors on lipoprotein lipase (LPL)**. Imperfections for 22 blocks from LPL. Each data point has an x-coordinate corresponding to the central SNP of a given block and a y-coordinate corresponding to the imperfection of the inferred phylogeny on that block. Data is shown for our direct method (solid grey line with squares), indirect inference with fastPHASE (dotted grey line with X's), indirect inference with haplotyper (dash-dot grey line with triangles), and the true imperfection (dashed black line with diamonds).

The results are shown in Figure 6, where the *x*-coordinate of each point is the central SNP of the block and the *y*-coordinate is the imperfection in that block. Most of the blocks are imperfect. On this dataset, in contrast to the prior ones, the direct and indirect approaches showed almost equal total accuracy, with haplotyper being slightly worse. This difference may reflect a failure to eliminate all recombination from the data set or might be because any advantage of direct inference is too modest to stand out on such a small data set. Even on a dataset that would be expected to be unusually easy for a phasing program, though, our method does no worse than the indirect approach. This dataset also suggests that the two approaches could be used in a complementary fashion, as the methods often bracket the true answer from opposite directions.

### Resource Usage

**Figure 7:**
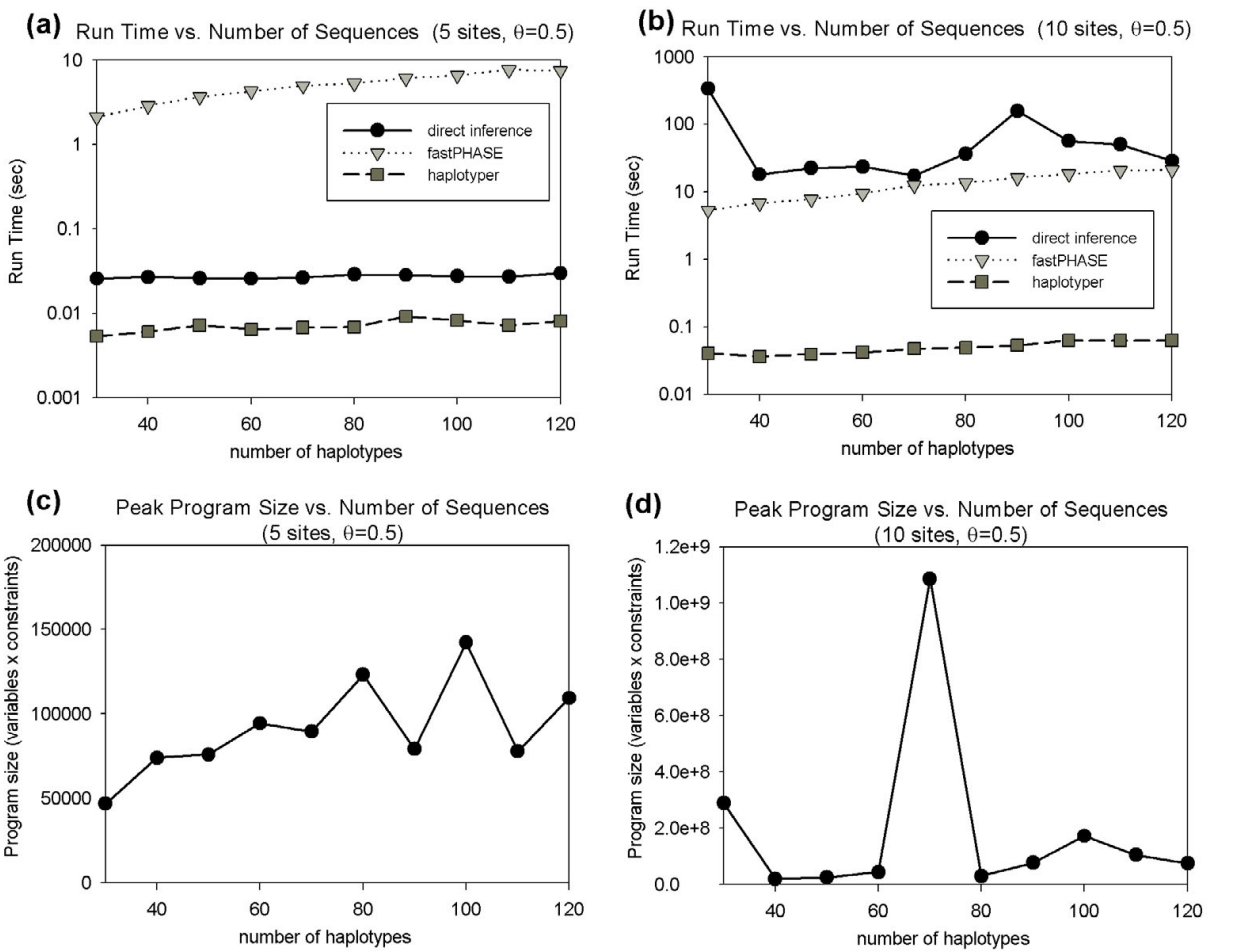
**Run time and space performance as a function of input size**. Each plot measures performance for fixed mutation rate *θ *- 0.5. Run time is measured in seconds processor time required per parameter value, averaged over 200 independent runs. Run time data is provided for our direct method (solid block line with circles), fastPHASE (dotted line with triangles), and haplotyper (dash-dot line with squares). Space usage is measured in maximum linear program size in variables × constraints over the full branch-and-bound execution, averaged over 200 independent runs, (a) Run time performance on 5-SNP windows, (b) Run time performance on 10-SNP windows, (c) Space usage on 5-SNP windows, (d) Space usage on 10-SNP windows.

We have, finally, examined the performance of our method in run time and space usage using additional simulation tests. We examined a range of data set sizes from 30 to 120 genotypes for fixed mutation rate *θ *= 0.5 for 5-SNP and 10-SNP windows using averages for 200 repetitions per parameter value. Run times were measured for our method and for fastPHASE and haplotyper. Figures 7(a) and 7(b) shows run times for the method for 5- and 10-SNP windows, respectively. Our method is consistently faster than fastPHASE and slower than haplotyper for 5-SNP windows. Like haplotyper and unlike fastPHASE, our method appears insensitive to the number of input sequences. Our method shows a substantial slowdown in moving from 5-SNP to 10-SNP windows. While the method is faster than fastPHASE for 5-SNP windows it is on average a few times slower with 10-SNP windows. This slowdown is to be expected since our method constructs a program of potentially exponential size in window size, haplotyper is consistently the fastest of the methods for both window sizes.

We further assessed space usage of our method based on the maximum linear program relaxation size examined over the course of a given problem instance, averaging this value over the 200 trials. Here size is expressed as the product of the number variables and constraints. Figures 7(c) and 7(d) show the results for 5- and 10-SNP windows. The results show a high degree of noise, with a single outlier point requiring roughly 100-fold more space than the others. Nonetheless, program size appears generally to increase with number of input sequences. Space usage also increases substantially with window size, which we would again expect given that worst-case program size is exponential in window size.

## Conclusion

We have developed the first practical, general methods for finding maximum parsimony haplotypes from unphased genotype data and have used them to assess the costs introduced by computational phasing prior to phylogenetic inference. Our methods used a collection of heuristics based on the theory of Steiner trees, a variant of a flow-based ILP, and a branch-and-bound approach to solve problem instances with high imperfection that were not solvable by any prior method. While the method presented here is specific to the problem of inferring purely mutational phylogenies, similar approaches may prove productive for inference of ancestry by more general models of molecular evolution, such as ancestral recombination graphs (ARGs). Empirical tests on simulated and semi-simulated data show that direct phylogeny inference from genotypes leads to fewer errors than does the standard practice of building phylogenies from phased data. Methods for this problem have several practical applications. Most important is to estimate the minimum number of recurrent mutations required to explain a set of observed genotypes. A large such value may indicate frequent recurrent mutation or gene conversion or a selective pressure to recurrently alter a given allele. Researchers trying to establish such effects need to ensure that the size of the phylogeny is not an artifact of phase inference. The method should similarly be useful for improving estimates of local mutation rates. Other applications include improving the power of association tests by eliminating spurious effects from recurrent mutation, and providing alternative methods for detecting recombination-free autosomal regions and performing phase inference from genotype data.

## Methods

We implemented two versions of the integer linear program both of which were competitive in practice. The first is a direct integer linear program implementation and the second is a branch-and-bound algorithm that wraps over a second integer linear program. We describe the direct implementation first followed by the branch-and-bound method. Both methods were implemented in C++ using the Concert Technology of CPLEX 10.0 for integer linear program (ILP) solutions. We found the branch-and-bound method to give generally lower run times in practice than the direct ILP method. We therefore used the branch-and-bound method exclusively in generating the empirical results presented here.

### Direct Integer Linear Programming Approach

This section introduces our ILP algorithm to solve the Genotype MP Phylogeny Problem. In the first subsection, we introduce pre-processing techniques that typically reduce the problem size after which we describe the ILP.

#### Preprocessing

Preprocessing techniques form an integral part of any solution method based on integer programming. We now describe the major preprocessing methods used.

Let *G *be the *n *× *m *input genotype matrix. Without loss of generality we can assume that all *m *sites are varying. We can also remove redundant rows (genotypes) of *G *until all rows are distinct, since this does not change the length of the optimal phylogeny. We now describe a method to remove redundant sites (columns) from *G*. Note that we are free to exchange labels 0 and 2 (homozygous major and minor alleles) independently at each site without change in the size of the phylogeny. Therefore two sites *i *and *j *are considered redundant if they are identical or become identical after relabeling one site.

For all sites *k*, let weight *w*_*k *_be initialized to 1. We then iteratively perform the following operation: for any pair of redundant sites *i*, *j*, set *w*_*i *_:= *w*_*i *_+ *w*_*j*_, and remove site *j *from the matrix. Let *G' *be the final matrix after this sequence of preprocessing steps. We now redefine the length of a phylogeny using a weighted Hamming distance as follows.

### Definition 3

*The *length *of phylogeny T*(*V*, *E*) *is *length(*T*) = ∑_(*u*,*v*)∈*E*_∑_*i*∈*D*(*u*,*v*)_*w*_*i*_, *where D*(*u*, *v*) *is the set of sites where u, v differ*.

The following lemma justifies the preprocessing step:

### Lemma 1

*The transformation from genotype matrix G to weighted genotype matrix G' does not change the length of the most parsimonious phylogeny*.

### Proof 1

*For any genotype matrix I, let T*_*I *_*denote the optimal phylogeny on I. For a site i of I, let j be a redundant site and consider the matrix I *∪ {*j*}. *The topology of phylogeny T*_*I *_*also gives a phylogeny for I *∪ {*j*}, *obtained by mutating j wherever i mutates. The length of T*_*I*∪{*j*} _*is *length(*T*_*I*∪{*j*}_) = length(*T*_*I*_) + *μ*(*i*), *where μ*(*i*) *is the number of times site i mutates in T*_*I*_. *Now, assume that the most parsimonious phylogeny T*_*G *_*for G resolves redundant genotype sites i and j differently, i.e., there is a haplotype for which sites i and j differ. Without loss of generality, suppose μ*(*i*) ≤ *μ*(*j*) *in T*_*G*_. *Then removing column j from T*_*G *_*results in a phylogeny T*_*G*\{*j*} _*with *length(*T*_*G*\{*j*}_) = length(*T*_*G*_) - *μ*(*j*). *Now, since j is identical to i, the argument above implies that adding site j back to the phylogeny gives a tree with length *length(*T*_*G*_) - *μ*(*j*) + *μ*(*i*) ≤ length(*T*_*G*_). *Therefore, there is an optimal phylogeny resolving sites i and j identically*.

Due to these preprocessing steps, we assume from now on that the input genotype matrix *G *has distinct rows, distinct sites, and weights *w*_*i *_≥ 1 associated to sites. For each genotype *g *∈ *G*, we create the set *R*(*g*) consisting of all possible pairs of haplotypes explaining *g*. Note that if *p *is the number of heterozygous sites in *g*, then *R*(*g*) consists of *2*^*p*-1 ^pairs of haplotypes.

Now, consider the matrix *H *= ⋃_*g*∈*G*_*R*(*g*), where the rows are all possible haplotypes that can explain input genotypes in *G*. *H *is a binary matrix, and for such instances, structural properties of the optimal phylogeny can be captured by a graph known as the *Buneman graph *ℬ
 MathType@MTEF@5@5@+=feaafiart1ev1aaatCvAUfKttLearuWrP9MDH5MBPbIqV92AaeXatLxBI9gBaebbnrfifHhDYfgasaacPC6xNi=xH8viVGI8Gi=hEeeu0xXdbba9frFj0xb9qqpG0dXdb9aspeI8k8fiI+fsY=rqGqVepae9pg0db9vqaiVgFr0xfr=xfr=xc9adbaqaaeGacaGaaiaabeqaaeqabiWaaaGcbaWenfgDOvwBHrxAJfwnHbqeg0uy0HwzTfgDPnwy1aaceaGae8hlHieaaa@36A4@(*H*) [30]. We will explain the generalization of this graph due to Barthélemy [31].

For a binary input matrix *H *and a site *c *of *H*, the *split c*(0)|*c*(1) defined by *c *is a partition of the haplotypes into two sets, where *c*(0) is the set of haplotypes with value 0 in site *c *and *c*(1) is the set of haplotypes with value 1 in site *c*. Each of *c*(0) and *c*(1) is called a *block *of *c*. Each vertex of the Buneman graph is an *m*-tuple of blocks [*c*_1_(*i*_1_), *c*_2_(*i*_2_),...*c*_*m*_(*i*_*m*_)] (*i*_*j *_= 0 or 1 for each 1 ≤ *j *≤ *m*), with one block for each site and such that each pair of blocks *c*_*j*_(*i*_*j*_) ∩ *c*_*k*_(*i*_*k*_) has nonempty intersection. There is an edge between two vertices in ℬ
 MathType@MTEF@5@5@+=feaafiart1ev1aaatCvAUfKttLearuWrP9MDH5MBPbIqV92AaeXatLxBI9gBaebbnrfifHhDYfgasaacPC6xNi=xH8viVGI8Gi=hEeeu0xXdbba9frFj0xb9qqpG0dXdb9aspeI8k8fiI+fsY=rqGqVepae9pg0db9vqaiVgFr0xfr=xfr=xc9adbaqaaeGacaGaaiaabeqaaeqabiWaaaGcbaWenfgDOvwBHrxAJfwnHbqeg0uy0HwzTfgDPnwy1aaceaGae8hlHieaaa@36A4@(*H*) if and only if they differ in exactly one block. Notice that vertices in the Buneman graph can be viewed simply as haplotypes. An *m*-tuple [*c*_1_(*i*_1_),...,*c*_*m*_(*i*_*m*_)] translates to haplotype (*i*_1_,...,*i*_*m*_). Buneman graphs are very useful due to the following theorem:

### Theorem 1

[3,5]*Let *ℬ
 MathType@MTEF@5@5@+=feaafiart1ev1aaatCvAUfKttLearuWrP9MDH5MBPbIqV92AaeXatLxBI9gBaebbnrfifHhDYfgasaacPC6xNi=xH8viVGI8Gi=hEeeu0xXdbba9frFj0xb9qqpG0dXdb9aspeI8k8fiI+fsY=rqGqVepae9pg0db9vqaiVgFr0xfr=xfr=xc9adbaqaaeGacaGaaiaabeqaaeqabiWaaaGcbaWenfgDOvwBHrxAJfwnHbqeg0uy0HwzTfgDPnwy1aaceaGae8hlHieaaa@36A4@(*H*) *be the Buneman graph for binary (haplotype) matrix H. Every optimal phylogeny *TH∗
 MathType@MTEF@5@5@+=feaafiart1ev1aaatCvAUfKttLearuWrP9MDH5MBPbIqV92AaeXatLxBI9gBaebbnrfifHhDYfgasaacPC6xNi=xH8viVGI8Gi=hEeeu0xXdbba9frFj0xb9qqpG0dXdb9aspeI8k8fiI+fsY=rqGqVepae9pg0db9vqaiVgFr0xfr=xfr=xc9adbaqaaeGacaGaaiaabeqaaeqabiWaaaGcbaGaemivaq1aa0baaSqaaiabdIeaibqaaiabgEHiQaaaaaa@2F39@*is a subgraph of *ℬ
 MathType@MTEF@5@5@+=feaafiart1ev1aaatCvAUfKttLearuWrP9MDH5MBPbIqV92AaeXatLxBI9gBaebbnrfifHhDYfgasaacPC6xNi=xH8viVGI8Gi=hEeeu0xXdbba9frFj0xb9qqpG0dXdb9aspeI8k8fiI+fsY=rqGqVepae9pg0db9vqaiVgFr0xfr=xfr=xc9adbaqaaeGacaGaaiaabeqaaeqabiWaaaGcbaWenfgDOvwBHrxAJfwnHbqeg0uy0HwzTfgDPnwy1aaceaGae8hlHieaaa@36A4@(*H*).

Using Theorem 1, we first construct the Buneman graph on *H *and then solve the phylogeny problem on underlying graph ℬ
 MathType@MTEF@5@5@+=feaafiart1ev1aaatCvAUfKttLearuWrP9MDH5MBPbIqV92AaeXatLxBI9gBaebbnrfifHhDYfgasaacPC6xNi=xH8viVGI8Gi=hEeeu0xXdbba9frFj0xb9qqpG0dXdb9aspeI8k8fiI+fsY=rqGqVepae9pg0db9vqaiVgFr0xfr=xfr=xc9adbaqaaeGacaGaaiaabeqaaeqabiWaaaGcbaWenfgDOvwBHrxAJfwnHbqeg0uy0HwzTfgDPnwy1aaceaGae8hlHieaaa@36A4@(*H*). The following lemma gives a bound on the time required to construct ℬ
 MathType@MTEF@5@5@+=feaafiart1ev1aaatCvAUfKttLearuWrP9MDH5MBPbIqV92AaeXatLxBI9gBaebbnrfifHhDYfgasaacPC6xNi=xH8viVGI8Gi=hEeeu0xXdbba9frFj0xb9qqpG0dXdb9aspeI8k8fiI+fsY=rqGqVepae9pg0db9vqaiVgFr0xfr=xfr=xc9adbaqaaeGacaGaaiaabeqaaeqabiWaaaGcbaWenfgDOvwBHrxAJfwnHbqeg0uy0HwzTfgDPnwy1aaceaGae8hlHieaaa@36A4@(*H*).

### Lemma 2

[11]*The Buneman graph *ℬ
 MathType@MTEF@5@5@+=feaafiart1ev1aaatCvAUfKttLearuWrP9MDH5MBPbIqV92AaeXatLxBI9gBaebbnrfifHhDYfgasaacPC6xNi=xH8viVGI8Gi=hEeeu0xXdbba9frFj0xb9qqpG0dXdb9aspeI8k8fiI+fsY=rqGqVepae9pg0db9vqaiVgFr0xfr=xfr=xc9adbaqaaeGacaGaaiaabeqaaeqabiWaaaGcbaWenfgDOvwBHrxAJfwnHbqeg0uy0HwzTfgDPnwy1aaceaGae8hlHieaaa@36A4@(*H*) *for input H on m sites can be constructed in time O(km) where k is the number of vertices in *ℬ
 MathType@MTEF@5@5@+=feaafiart1ev1aaatCvAUfKttLearuWrP9MDH5MBPbIqV92AaeXatLxBI9gBaebbnrfifHhDYfgasaacPC6xNi=xH8viVGI8Gi=hEeeu0xXdbba9frFj0xb9qqpG0dXdb9aspeI8k8fiI+fsY=rqGqVepae9pg0db9vqaiVgFr0xfr=xfr=xc9adbaqaaeGacaGaaiaabeqaaeqabiWaaaGcbaWenfgDOvwBHrxAJfwnHbqeg0uy0HwzTfgDPnwy1aaceaGae8hlHieaaa@36A4@(*H*).

The Buneman graph is simply a method to reduce the size of the underlying graph from an *m*-cube with 2^*m *^vertices to a (typically significantly) smaller sub-graph. Putting together these methods, we can summarize our preprocessing steps as follows:

1. Create a weighted genotype matrix *G *where sites are pair-wise distinct.

2. Create a set *H *of all possible haplotypes explaining rows of *G*.

3. Construct the underlying graph *F*(*V*, *E*) = ℬ
 MathType@MTEF@5@5@+=feaafiart1ev1aaatCvAUfKttLearuWrP9MDH5MBPbIqV92AaeXatLxBI9gBaebbnrfifHhDYfgasaacPC6xNi=xH8viVGI8Gi=hEeeu0xXdbba9frFj0xb9qqpG0dXdb9aspeI8k8fiI+fsY=rqGqVepae9pg0db9vqaiVgFr0xfr=xfr=xc9adbaqaaeGacaGaaiaabeqaaeqabiWaaaGcbaWenfgDOvwBHrxAJfwnHbqeg0uy0HwzTfgDPnwy1aaceaGae8hlHieaaa@36A4@(*H*) where *H *⊆ *V *and (*u*, *v*) ∈ *E *connects two vertices (haplotypes) if and only if they differ in exactly one site. Edge weights *w*_*u*,*v *_= *w*_*i *_where *i *is the site at which *u *and *v *differ.

We apply some additional heuristic preprocessing steps that have proven very effective in practice. One of these steps identifies a subset of haplotypes that must occur in any optimal solution and then removes from the input any genotypes that can be produced from pairs of these obligatory haplotypes. As any optimal output can produce these genotypes, their absence will not change the final output. We can also eliminate certain possible haplotypes because they would imply high-weight edges and therefore cannot occur in any low-cost solution.

Once all preprocessing steps have been applied, we have a weighted Buneman graph *B*(*H*) that contains every node and edge that might be included in any optimal phylogeny for *G*. We now show an ILP formulation to simultaneously select the optimal subset *H' *⊆ *H *such that all of *G *can be derived from *H' *and connect *H' *using a tree.

#### ILP Formulation

We now develop an ILP formulation for the problem based on multicommodity flows [32]. The formulation borrows from prior work on fast ILP solution of maximum parsimony phylogenies on haplotypes [11]. Although this formulation can use exponential numbers of variables and constraints in the worst case, it is fast in practice. It is possible to solve the maximum parsimony genotype problem using an ILP with polynomial numbers of variables and constraints, but all polynomial-size variants that we developed proved intractable in practice.

The high-level idea of the method is to send flow from a designated root to each haplotype that is used to explain an input genotype. Each of these haplotypes acts as a sink for one unit of flow. The program must select a subset of edges that accommodate all flow while minimizing the cost of the edges selected. This flow formulation guarantees that every haplotype is connected to the root and the minimization prevents formation of cycles. The formulation thus forces the output to be a tree. For the sake of simplicity, we assume that the all-zeros haplotype is present in all the solutions. We can treat this as the *root*.

Let *h*_*k *_be an indicator variable denoting the presence or absence of haplotype *k *∈ *H*. If *h*_*k *_= 1, *k *is called a *present *haplotype. We have binary variables *p*_*i*,*j *_that denote the presence of both haplotypes *h*_*i *_and *h*_*j*_. All the present haplotypes act as a sink for one unit of flow from the root vertex. On the other hand, all non-present haplotype vertices and Steiner vertices satisfy perfect flow conservation. To enforce this, we use two types of binary variables fi,jk
 MathType@MTEF@5@5@+=feaafiart1ev1aaatCvAUfKttLearuWrP9MDH5MBPbIqV92AaeXatLxBI9gBaebbnrfifHhDYfgasaacPC6xNi=xH8viVGI8Gi=hEeeu0xXdbba9frFj0xb9qqpG0dXdb9aspeI8k8fiI+fsY=rqGqVepae9pg0db9vqaiVgFr0xfr=xfr=xc9adbaqaaeGacaGaaiaabeqaaeqabiWaaaGcbaGaemOzay2aa0baaSqaaiabdMgaPjabcYcaSiabdQgaQbqaaiabdUgaRbaaaaa@324C@ and *s*_*i*,*j *_for each edge (*i*, *j*) ∈ *E*. The variables fi,jk
 MathType@MTEF@5@5@+=feaafiart1ev1aaatCvAUfKttLearuWrP9MDH5MBPbIqV92AaeXatLxBI9gBaebbnrfifHhDYfgasaacPC6xNi=xH8viVGI8Gi=hEeeu0xXdbba9frFj0xb9qqpG0dXdb9aspeI8k8fiI+fsY=rqGqVepae9pg0db9vqaiVgFr0xfr=xfr=xc9adbaqaaeGacaGaaiaabeqaaeqabiWaaaGcbaGaemOzay2aa0baaSqaaiabdMgaPjabcYcaSiabdQgaQbqaaiabdUgaRbaaaaa@324C@ are real valued and represent the amount of flow along edge (*i*, *j*) whose final destination is haplotype *k*. Note that if *k *is a non-present haplotype, then fi,jk
 MathType@MTEF@5@5@+=feaafiart1ev1aaatCvAUfKttLearuWrP9MDH5MBPbIqV92AaeXatLxBI9gBaebbnrfifHhDYfgasaacPC6xNi=xH8viVGI8Gi=hEeeu0xXdbba9frFj0xb9qqpG0dXdb9aspeI8k8fiI+fsY=rqGqVepae9pg0db9vqaiVgFr0xfr=xfr=xc9adbaqaaeGacaGaaiaabeqaaeqabiWaaaGcbaGaemOzay2aa0baaSqaaiabdMgaPjabcYcaSiabdQgaQbqaaiabdUgaRbaaaaa@324C@ should be set to 0 for all edges (*i*, *j*). Variables *s*_*i*,*j *_are binary variables that denotes if edge (*i*, *j*) of the graph has been selected. We want to enforce that flow can be sent along edge (*i*, *j*) only if it has been selected.

We now have the following integer linear program:

min   ∑_*i*,*j*_*w*_*i*,*j*_*s*_*i*,*j*_

s.t.   *p*_*ij *_≤ *h*_*i*_   ∀*i*, *j *∈ *H*

*p*_*ij *_≤ *h*_*j*_   ∀*i*, *j *∈ *H*

∑_(*i*,*j*)∈*R*(*g*)_*p*_*ij *_≥ 1   ∀input *g *∈ *G*

∑jf0,jk=∑jfj,kk=hk∀k∈H
 MathType@MTEF@5@5@+=feaafiart1ev1aaatCvAUfKttLearuWrP9MDH5MBPbIqV92AaeXatLxBI9gBaebbnrfifHhDYfgasaacPC6xNi=xI8qiVKYPFjYdHaVhbbf9v8qqaqFr0xc9vqFj0dXdbba91qpepeI8k8fiI+fsY=rqGqVepae9pg0db9vqaiVgFr0xfr=xfr=xc9adbaqaaeGacaGaaiaabeqaaeqabiWaaaGcbaqbaeqabeGaaaqaamaaqababaGaemOzay2aa0baaSqaaiabicdaWiabcYcaSiabdQgaQbqaaiabdUgaRbaakiabg2da9aWcbaGaemOAaOgabeqdcqGHris5aOWaaabeaeaacqWGMbGzdaqhaaWcbaGaemOAaOMaeiilaWIaem4AaSgabaGaem4AaSgaaaqaaiabdQgaQbqab0GaeyyeIuoakiabg2da9iabdIgaOnaaBaaaleaacqWGRbWAaeqaaaGcbaGaeyiaIiIaem4AaSMaeyicI4SaemisaGeaaaaa@4910@

∑jfi,jk=∑jfj,ik∀i≠0,k,k∈H
 MathType@MTEF@5@5@+=feaafiart1ev1aaatCvAUfKttLearuWrP9MDH5MBPbIqV92AaeXatLxBI9gBaebbnrfifHhDYfgasaacPC6xNi=xI8qiVKYPFjYdHaVhbbf9v8qqaqFr0xc9vqFj0dXdbba91qpepeI8k8fiI+fsY=rqGqVepae9pg0db9vqaiVgFr0xfr=xfr=xc9adbaqaaeGacaGaaiaabeqaaeqabiWaaaGcbaqbaeqabeGaaaqaamaaqababaGaemOzay2aa0baaSqaaiabdMgaPjabcYcaSiabdQgaQbqaaiabdUgaRbaakiabg2da9aWcbaGaemOAaOgabeqdcqGHris5aOWaaabeaeaacqWGMbGzdaqhaaWcbaGaemOAaOMaeiilaWIaemyAaKgabaGaem4AaSgaaaqaaiabdQgaQbqab0GaeyyeIuoaaOqaaiabgcGiIiabdMgaPjabgcMi5kabicdaWiabcYcaSiabdUgaRjabcYcaSiabdUgaRjabgIGiolabdIeaibaaaaa@4CB4@

fi,jk≤si,j∀i,j,k
 MathType@MTEF@5@5@+=feaafiart1ev1aaatCvAUfKttLearuWrP9MDH5MBPbIqV92AaeXatLxBI9gBaebbnrfifHhDYfgasaacPC6xNi=xI8qiVKYPFjYdHaVhbbf9v8qqaqFr0xc9vqFj0dXdbba91qpepeI8k8fiI+fsY=rqGqVepae9pg0db9vqaiVgFr0xfr=xfr=xc9adbaqaaeGacaGaaiaabeqaaeqabiWaaaGcbaqbaeqabeGaaaqaaiabdAgaMnaaDaaaleaacqWGPbqAcqGGSaalcqWGQbGAaeaacqWGRbWAaaGccqGHKjYOcqWGZbWCdaWgaaWcbaGaemyAaKMaeiilaWIaemOAaOgabeaaaOqaaiabgcGiIiabdMgaPjabcYcaSiabdQgaQjabcYcaSiabdUgaRbaaaaa@404A@

In constraints (2) and (3), variable *p*_*ij *_indicates the presence of the haplotype pair (*h*_*i*_, *h*_*j*_). Constraint (4) guarantees that each genotype is explained by at least one pair of haplotypes. Constraint (5) imposes inflow/outflow constraints on haplotypes as well as enforcing the condition that there is positive flow to a haplotype *h*_*k *_only if *h*_*k *_is selected. Constraint (6) imposes flow conservation at all non-present haplotype vertices as well as Steiner vertices and constraint (7) imposes the condition that flow can only be sent along edges present in the solution. Note that all integer variables of the above linear program are binary. Finally, we observe that the solution of the ILP is the size of the most parsimonious phylogeny on *G*.

### Branch and Bound Algorithm

We developed an alternative method for the problem that uses a simpler integer linear program embedded in a branch-and-bound routine. The high-level idea behind the method is to first guess the set of haplotypes that would phase the given input genotypes and then construct a most parsimonious phylogeny on the haplotypes. Note that all the pre-processing techniques outlined in the previous sub-section still apply for this method.

We use *G *to refer to the input set of genotypes. For a given set of haplotypes ℋ
 MathType@MTEF@5@5@+=feaafiart1ev1aaatCvAUfKttLearuWrP9MDH5MBPbIqV92AaeXatLxBI9gBaebbnrfifHhDYfgasaacPC6xNi=xH8viVGI8Gi=hEeeu0xXdbba9frFj0xb9qqpG0dXdb9aspeI8k8fiI+fsY=rqGqVepae9pg0db9vqaiVgFr0xfr=xfr=xc9adbaqaaeGacaGaaiaabeqaaeqabiWaaaGcbaWenfgDOvwBHrxAJfwnHbqeg0uy0HwzTfgDPnwy1aaceaGae83cHGeaaa@3689@, we can construct the most parsimonious phylogeny Tℋ
 MathType@MTEF@5@5@+=feaafiart1ev1aaatCvAUfKttLearuWrP9MDH5MBPbIqV92AaeXatLxBI9gBaebbnrfifHhDYfgasaacPC6xNi=xH8viVGI8Gi=hEeeu0xXdbba9frFj0xb9qqpG0dXdb9aspeI8k8fiI+fsY=rqGqVepae9pg0db9vqaiVgFr0xfr=xfr=xc9adbaqaaeGacaGaaiaabeqaaeqabiWaaaGcbaGaemivaq1aaSbaaSqaamrtHrhAL1wy0L2yHvtyaeHbnfgDOvwBHrxAJfwnaGabaiab=Tqiibqabaaaaa@37E6@ using the algorithm described by Sridhar et al. [33]. We will use hapMP to denote this algorithm, which will take a set of haplotypes and return the size of the most parsimonious phylogeny. We now have the following branch and bound method.

function genBB(genotypes *G*, haplotypes ℋ
 MathType@MTEF@5@5@+=feaafiart1ev1aaatCvAUfKttLearuWrP9MDH5MBPbIqV92AaeXatLxBI9gBaebbnrfifHhDYfgasaacPC6xNi=xH8viVGI8Gi=hEeeu0xXdbba9frFj0xb9qqpG0dXdb9aspeI8k8fiI+fsY=rqGqVepae9pg0db9vqaiVgFr0xfr=xfr=xc9adbaqaaeGacaGaaiaabeqaaeqabiWaaaGcbaWenfgDOvwBHrxAJfwnHbqeg0uy0HwzTfgDPnwy1aaceaGae83cHGeaaa@3689@, integer *u*)

   1. for all row vectors g→
 MathType@MTEF@5@5@+=feaafiart1ev1aaatCvAUfKttLearuWrP9MDH5MBPbIqV92AaeXatLxBI9gBaebbnrfifHhDYfgasaacPC6xNi=xH8viVGI8Gi=hEeeu0xXdbba9frFj0xb9qqpG0dXdb9aspeI8k8fiI+fsY=rqGqVepae9pg0db9vqaiVgFr0xfr=xfr=xc9adbaqaaeGacaGaaiaabeqaaeqabiWaaaGcbaGafm4zaCMbaSaaaaa@2D3C@ ∈ *G*

      (a) if ∃*h*_1_, *h*_2 _∈ ℋ
 MathType@MTEF@5@5@+=feaafiart1ev1aaatCvAUfKttLearuWrP9MDH5MBPbIqV92AaeXatLxBI9gBaebbnrfifHhDYfgasaacPC6xNi=xH8viVGI8Gi=hEeeu0xXdbba9frFj0xb9qqpG0dXdb9aspeI8k8fiI+fsY=rqGqVepae9pg0db9vqaiVgFr0xfr=xfr=xc9adbaqaaeGacaGaaiaabeqaaeqabiWaaaGcbaWenfgDOvwBHrxAJfwnHbqeg0uy0HwzTfgDPnwy1aaceaGae83cHGeaaa@3689@ s.t. h→1+h→2=g→
 MathType@MTEF@5@5@+=feaafiart1ev1aaatCvAUfKttLearuWrP9MDH5MBPbIqV92AaeXatLxBI9gBaebbnrfifHhDYfgasaacPC6xNi=xH8viVGI8Gi=hEeeu0xXdbba9frFj0xb9qqpG0dXdb9aspeI8k8fiI+fsY=rqGqVepae9pg0db9vqaiVgFr0xfr=xfr=xc9adbaqaaeGacaGaaiaabeqaaeqabiWaaaGcbaGafmiAaGMbaSaadaWgaaWcbaGaeGymaedabeaakiabgUcaRiqbdIgaOzaalaWaaSbaaSqaaiabikdaYaqabaGccqGH9aqpcuWGNbWzgaWcaaaa@3448@ then *G *← *G*\{*g*}

   2. if (|*G*| = ∅) then return hapMP(ℋ
 MathType@MTEF@5@5@+=feaafiart1ev1aaatCvAUfKttLearuWrP9MDH5MBPbIqV92AaeXatLxBI9gBaebbnrfifHhDYfgasaacPC6xNi=xH8viVGI8Gi=hEeeu0xXdbba9frFj0xb9qqpG0dXdb9aspeI8k8fiI+fsY=rqGqVepae9pg0db9vqaiVgFr0xfr=xfr=xc9adbaqaaeGacaGaaiaabeqaaeqabiWaaaGcbaWenfgDOvwBHrxAJfwnHbqeg0uy0HwzTfgDPnwy1aaceaGae83cHGeaaa@3689@)

   3. if (hapMP(ℋ
 MathType@MTEF@5@5@+=feaafiart1ev1aaatCvAUfKttLearuWrP9MDH5MBPbIqV92AaeXatLxBI9gBaebbnrfifHhDYfgasaacPC6xNi=xH8viVGI8Gi=hEeeu0xXdbba9frFj0xb9qqpG0dXdb9aspeI8k8fiI+fsY=rqGqVepae9pg0db9vqaiVgFr0xfr=xfr=xc9adbaqaaeGacaGaaiaabeqaaeqabiWaaaGcbaWenfgDOvwBHrxAJfwnHbqeg0uy0HwzTfgDPnwy1aaceaGae83cHGeaaa@3689@) ≥ *u *- 1) then return ∞

   4. let g→
 MathType@MTEF@5@5@+=feaafiart1ev1aaatCvAUfKttLearuWrP9MDH5MBPbIqV92AaeXatLxBI9gBaebbnrfifHhDYfgasaacPC6xNi=xH8viVGI8Gi=hEeeu0xXdbba9frFj0xb9qqpG0dXdb9aspeI8k8fiI+fsY=rqGqVepae9pg0db9vqaiVgFr0xfr=xfr=xc9adbaqaaeGacaGaaiaabeqaaeqabiWaaaGcbaGafm4zaCMbaSaaaaa@2D3C@ be a row vector of *G*

   5. for all h→1
 MathType@MTEF@5@5@+=feaafiart1ev1aaatCvAUfKttLearuWrP9MDH5MBPbIqV92AaeXatLxBI9gBaebbnrfifHhDYfgasaacPC6xNi=xH8viVGI8Gi=hEeeu0xXdbba9frFj0xb9qqpG0dXdb9aspeI8k8fiI+fsY=rqGqVepae9pg0db9vqaiVgFr0xfr=xfr=xc9adbaqaaeGacaGaaiaabeqaaeqabiWaaaGcbaGafmiAaGMbaSaadaWgaaWcbaGaeGymaedabeaaaaa@2E5A@, h→2
 MathType@MTEF@5@5@+=feaafiart1ev1aaatCvAUfKttLearuWrP9MDH5MBPbIqV92AaeXatLxBI9gBaebbnrfifHhDYfgasaacPC6xNi=xH8viVGI8Gi=hEeeu0xXdbba9frFj0xb9qqpG0dXdb9aspeI8k8fiI+fsY=rqGqVepae9pg0db9vqaiVgFr0xfr=xfr=xc9adbaqaaeGacaGaaiaabeqaaeqabiWaaaGcbaGafmiAaGMbaSaadaWgaaWcbaGaeGOmaidabeaaaaa@2E5C@ s.t. h→1+h→2=g→
 MathType@MTEF@5@5@+=feaafiart1ev1aaatCvAUfKttLearuWrP9MDH5MBPbIqV92AaeXatLxBI9gBaebbnrfifHhDYfgasaacPC6xNi=xH8viVGI8Gi=hEeeu0xXdbba9frFj0xb9qqpG0dXdb9aspeI8k8fiI+fsY=rqGqVepae9pg0db9vqaiVgFr0xfr=xfr=xc9adbaqaaeGacaGaaiaabeqaaeqabiWaaaGcbaGafmiAaGMbaSaadaWgaaWcbaGaeGymaedabeaakiabgUcaRiqbdIgaOzaalaWaaSbaaSqaaiabikdaYaqabaGccqGH9aqpcuWGNbWzgaWcaaaa@3448@

      (a) *G' *← *G*\{g→
 MathType@MTEF@5@5@+=feaafiart1ev1aaatCvAUfKttLearuWrP9MDH5MBPbIqV92AaeXatLxBI9gBaebbnrfifHhDYfgasaacPC6xNi=xH8viVGI8Gi=hEeeu0xXdbba9frFj0xb9qqpG0dXdb9aspeI8k8fiI+fsY=rqGqVepae9pg0db9vqaiVgFr0xfr=xfr=xc9adbaqaaeGacaGaaiaabeqaaeqabiWaaaGcbaGafm4zaCMbaSaaaaa@2D3C@}

      (b) *H' *← ℋ
 MathType@MTEF@5@5@+=feaafiart1ev1aaatCvAUfKttLearuWrP9MDH5MBPbIqV92AaeXatLxBI9gBaebbnrfifHhDYfgasaacPC6xNi=xH8viVGI8Gi=hEeeu0xXdbba9frFj0xb9qqpG0dXdb9aspeI8k8fiI+fsY=rqGqVepae9pg0db9vqaiVgFr0xfr=xfr=xc9adbaqaaeGacaGaaiaabeqaaeqabiWaaaGcbaWenfgDOvwBHrxAJfwnHbqeg0uy0HwzTfgDPnwy1aaceaGae83cHGeaaa@3689@ ∪ {*h*_1_, *h*_2_}

      (c) *b *← genBB(*G'*, *H'*, *u*)

      (d) if *b *<*u *then *u *← *b*

   6. return *u*

The *branch step *is performed by Step 5, where the algorithm attempts to phase genotype *g *using all possible pairs of haplotypes *h*_1_, *h*_2_. Integer *u *of the above pseudo-code refers to the current best upper-bound. The *bound step *is performed by Step 3 which just reconstructs a phylogeny over the current set ℋ
 MathType@MTEF@5@5@+=feaafiart1ev1aaatCvAUfKttLearuWrP9MDH5MBPbIqV92AaeXatLxBI9gBaebbnrfifHhDYfgasaacPC6xNi=xH8viVGI8Gi=hEeeu0xXdbba9frFj0xb9qqpG0dXdb9aspeI8k8fiI+fsY=rqGqVepae9pg0db9vqaiVgFr0xfr=xfr=xc9adbaqaaeGacaGaaiaabeqaaeqabiWaaaGcbaWenfgDOvwBHrxAJfwnHbqeg0uy0HwzTfgDPnwy1aaceaGae83cHGeaaa@3689@ of haplotypes. Step la ensures that at least one more haplotype *h *∉ ℋ
 MathType@MTEF@5@5@+=feaafiart1ev1aaatCvAUfKttLearuWrP9MDH5MBPbIqV92AaeXatLxBI9gBaebbnrfifHhDYfgasaacPC6xNi=xH8viVGI8Gi=hEeeu0xXdbba9frFj0xb9qqpG0dXdb9aspeI8k8fiI+fsY=rqGqVepae9pg0db9vqaiVgFr0xfr=xfr=xc9adbaqaaeGacaGaaiaabeqaaeqabiWaaaGcbaWenfgDOvwBHrxAJfwnHbqeg0uy0HwzTfgDPnwy1aaceaGae83cHGeaaa@3689@ is required to obtain the final set of haplotypes. Therefore, even if hapMP(ℋ
 MathType@MTEF@5@5@+=feaafiart1ev1aaatCvAUfKttLearuWrP9MDH5MBPbIqV92AaeXatLxBI9gBaebbnrfifHhDYfgasaacPC6xNi=xH8viVGI8Gi=hEeeu0xXdbba9frFj0xb9qqpG0dXdb9aspeI8k8fiI+fsY=rqGqVepae9pg0db9vqaiVgFr0xfr=xfr=xc9adbaqaaeGacaGaaiaabeqaaeqabiWaaaGcbaWenfgDOvwBHrxAJfwnHbqeg0uy0HwzTfgDPnwy1aaceaGae83cHGeaaa@3689@) = *u *- 1, this branch cannot yield a solution of smaller cost than current upper-bound *u*.

In the above method, the height of the branch-and-bound tree is at most *n*, the number of input genotypes. The branching factor at each internal node is at most 2^*k *^where *k *is the number of heterozygous sites on the genotype *g*. This is always bounded by 2^*m*^. Although the running-time of the final branch-and-bound method is super-exponential, we find that its run time is competitive with and often superior to the ILP described in the previous section.

### Data Generation and Analysis

In order to generate simulated data, coalescent trees were created using Hudson's ms program [34]. The only parameter required to generate tree topologies is the number of haploid chromosomes *n*_*h*_. The ms program can also use this tree to produce haplotype sequences, but does so under the infinite-sites model (without any recurrent mutations). We therefore instead used the seq-gen program of Rambaut and Grassly [35] to generate *n*_*h *_haplotypes using the ms coalescent tree. We varied the number of SNPs *m *and the mutation rate parameter *θ *= 4*N*_0_*μ*, where *μ *is the probability of mutation of any of the simulated SNPs in one generation and *N*_0 _is the effective population size. We relate the simulation parameter *μ *to the per-site mutation rate by assuming an effective population size *N*_0 _= 10, 000 (a reasonable estimate for humans [36]). For instance, for 5 sites, we obtain a per-site mutation probability of 10^-6 ^for *θ *= 0.2.

seq-gen was used under the GTR model, a generic time reversible Markov model. Mutation rates between *A *and *C *and between *G *and *T *were defined using the same parameter *θ*. For all the other four pairs we set the mutation rate to be 0 in order to produce biallelic data. The exact command line used to execute seq-gen for a given mutation rate parameter *θ *and SNP number *m *was the following:

seq-gen -mGTR -r *θ*, 0, 0, 0, 0, *θ *-l *m*

Each data point was generated from 200 independently generated simulated data sets, with the reported error rates summed over the 200 replicates. In our first set of simulations, designed to test the effect of mutation rate on accuracy, we varied *θ *over the range 0.2–0.6 in increments of 0.05 for windows of 5 and 10 SNPs and for sample sizes of 30 and 60 input haplotypes. Our second set of experiments, designed to test the effect of sample size on accuracy, fixed *θ *at 0.5 and varied the number of haplotypes from 30 to 120 in increments of 10 for windows of 5 and 10 SNPs. Data points plotted represent summed errors over the 200 replicates per parameter value.

Mitochondrial data was extracted from of a set 63 complete mitochondrial DNA sequences of 16,569 bases each produced by from Fraumene et al. [27]. We produced artificial diploids from the data by randomly selecting 60 of the sequences and randomly grouping them into 30 pairs. We computationally inferred haplotypes from all of the genotypes using fastPHASE and we constructed phylogenies for all sliding windows of 50 bases across the data set by each of three methods: maximum parsimony using true haplotypes, inferred haplotypes and from the genotypes.

Autosomal DNA was extracted from a lipoprotein lipase (LPL) data set due to Nickerson et al. [29]. Because the pairs of haplotypes into genotypes were not published, we duplicated the first haplotype to obtain 78 distinct sequences and then randomly paired them to produce 39 artificial genotypes from the true haplotypes. As in the previous case, we ran fastPHASE and haplotyper on all of the SNPs put together to obtain inferred haplotypes. In order to reduce the possibility of recombination events confounding our results, we used the HAP webserver [16] to break the 86 SNPs into blocks. HAP was also used to infer missing data. We then evaluated phylogeny sizes by our direct method, from the true haplotypes, and from the inferred haplotypes for each block.

## Availability and requirements

Project name: Direct Imperfect Phylogeny Reconstruction from Genotypes

**Project home page: **

The implementation of the algorithm that was used in our empirical study is accessible through a web form at the project web page. Instructions are provided at the site. Requirements below are for use of this web server. Source code in C ++ will be provided upon request, but requires that the user have access to ILOG CPLEX 10 and a CPLEX-supported operating system and compiler.

**Operating system(s): **Linux Redhat, Windows, Mac OS X

**Other requirements: **Web browser: software has been tested on Mozilla 1.6, Firefox 2.0.0.4, Internet Explorer 6.0, Internet Explorer Mac 5.2, and Safari 2.0.4.

**Any restrictions to non-academics: **Web-based access to the analysis tools is freely available.

## Authors' contributions

S.S. developed the software used in the paper and performed all the experiments. All authors participated in the design of the algorithms and suggesting methods for validation. All authors read and approved the final manuscript.
